# Oral administration of cystine and theanine attenuates 5-fluorouracil-induced intestinal mucositis and diarrhea by suppressing both glutathione level decrease and ROS production in the small intestine of mucositis mouse model

**DOI:** 10.1186/s12885-021-09057-z

**Published:** 2021-12-18

**Authors:** Junya Yoneda, Sachiko Nishikawa, Shigekazu Kurihara

**Affiliations:** Research Institute For Bioscience Products & Fine Chemicals Ajinomoto Co., Inc., 1-1, Suzuki-Cho, Kawasaki-ku, Kawasaki-shi, 210-8681 Japan

**Keywords:** Mucositis, Chemotherapy, Adverse events, Diarrhea, Cancer, Cystine/Theanine

## Abstract

**Background:**

Chemotherapy is frequently used in cancer treatment; however, it may cause adverse events, which must be managed. Reactive oxygen species (ROS) have been reported to be involved in the induction of intestinal mucositis and diarrhea, which are common side effects of treatment with fluoropyrimidine 5-fluorouracil (5-FU). Our previous studies have shown that oral administration of cystine and theanine (CT) increases glutathione (GSH) production in vivo. In the present study, we hypothesized that CT might inhibit oxidative stress, including the overproduction of ROS, and attenuate 5-FU-induced mucositis and diarrhea.

**Methods:**

We investigated the inhibitory effect of CT administration on mucositis and diarrhea, as well as its mechanism, using a mouse model of 5-FU-induced intestinal mucositis.

**Results:**

CT administration suppressed 5-FU-induced diarrhea and weight loss in the studied mice. After 5-FU administration, the GSH level and the GSH/GSSG ratio in the small intestine mucosal tissue decreased compared to normal control group; but CT administration improved the GSH/GSSG ratio to normal control levels. 5-FU induced ROS production in the basal region of the crypt of the small intestine mucosal tissue, which was inhibited by CT. CT did not affect the antitumor effect of 5-FU.

**Conclusions:**

CT administration suppressed intestinal mucositis and diarrhea in a mouse model. This finding might be associated with the antioxidant characteristics of CT, including the improved rate of GSH redox and the reduced rate of ROS production in the small intestine mucosal tissue. CT might be a suitable candidate for the treatment of gastrointestinal mucositis associated with chemotherapy.

**Supplementary Information:**

The online version contains supplementary material available at 10.1186/s12885-021-09057-z.

## Background

Although chemotherapy is an effective treatment for several types of cancer, it is also associated with a relatively high risk of adverse events [1]. Many types of anticancer drugs induce cancer cell death by inhibiting DNA and/or RNA synthesis, which suppresses cancer cell proliferation; as the rate of cancer cell division is higher than that of normal cells, chemotherapy can be selectively attack the former while sparing the latter. However, some normal tissue cells, for example, the oral and intestinal mucosa, also divide frequently and are thus more likely to be affected by the activity of anticancer drugs, resulting in the development of adverse events such as mucositis [2].

Patients with gastrointestinal mucositis tend to develop symptoms such as abdominal pain, malabsorption, vomiting, diarrhea, ulceration, and hemorrhage [1]. Among them, diarrhea interferes with nutrient and water absorption, induces electrolyte imbalance, malnutrition, and dehydration, which reduce patients’ quality of life and increase the risk of infection and sepsis. These symptoms can lead to chemotherapy dose reduction or discontinuation, potentially reducing treatment efficacy and increasing the socioeconomic cost of treatment due to its prolongation and associated hospitalization [3].

The fluoropyrimidine 5-fluorouracil (5-FU) is commonly used in the treatment of several cancer types, including head and neck, breast, prostate, pancreas, and liver cancers as well as cancers of the genitourinary and gastrointestinal systems [4].

The mechanism of 5-FU cytotoxicity has been implicated in the induction of apoptosis by inhibition of thymidylate synthase (TS) and incorporation of its metabolites into RNA and DNA [5]. Concurrently, 5-FU-induced intestinal mucositis is among the most common side effects of chemotherapy; in fact, 5-FU-induced oxidative stress, including reactive oxygen species (ROS) production [6], and the associated inflammation are risk factors for mucositis [7].

Cystine-theanine (CT) is a combination of the two amino acids at a weight ratio of 5:2. Cystine is a sulfur-containing amino acid, composed of two cysteine molecules linked via disulfide bonds. Cystine is reduced intracellularly and converted to cysteine. Meanwhile, theanine (g-glutamylethylamide), an amino acid abundant in green tea, is metabolized to glutamic acid and ethylamine in the intestine or liver [8]. Cysteine and glutamic acid are constituents of glutathione (GSH), the most abundant type of non-protein thiol that protects healthy tissues against the effects of oxidative stress [9]. Studies have shown that the availability of cysteine is the key determinant of the rate of GSH synthesis [10, 11]; in addition, CT intake has been shown to promote GSH biosynthesis [12].

Furthermore, GSH is an important determinant of redox signaling and has been shown to be essential for the detoxification of foreign bodies. In addition, it has been shown to regulate the rate of cell proliferation and apoptosis, and to contribute to the immune function and fibrosis [13].

Oxidative stress is defined as the imbalance between the rate of oxidant production and elimination [14]. GSH is a major cellular non-enzymatic antioxidant. The oxidation of GSH to disulfide (GSSG), followed by a subsequent decrease in the level of coupled GSH-GSSG is a useful indicator of cellular oxidative stress [15]. The GSH/GSSG redox state governs cell transition from the quiescent state to the proliferative state as well as growth arrest, differentiation, and apoptosis [16]. In addition, if the redox environment is highly oxidized, it promotes apoptosis or necrosis [17].

In our previous human study, we have shown that CT administration may suppress the increase and decrease in the number of neutrophils and lymphocytes, respectively, that result from heavy exercise in athletes [18]. CT also had a tendency to reduce the incidence rate of side effects, including diarrhea and hand foot syndrome (HFS) induced by capecitabine treatment in colorectal cancer patients [19]. Using a surgical mouse model, we have also shown that perioperative CT administration may suppress inflammatory responses by inhibiting the surgically induced decrease in GSH biosynthesis in the small intestine, thus promoting recovery [20, 21].

Based on these findings, we hypothesized that CT administration could suppress the 5-FU-induced intestinal mucositis and diarrhea by limiting the decrease in GSH levels in the intestinal tissue and reducing oxidative stress, including ROS production. In this study, we investigated the inhibitory effect of CT on 5-FU-induced mucositis and the resulting diarrhea, using the mouse model of 5-FU-induced enteromucositis.

## Methods

### Animals

Animals: Six-week-old male BALB/c mice (Charles River Japan, Inc.) were housed under specific pathogen-free conditions with food and water ad libitum, and were kept on a 12/12-h light/dark cycle. The mice were given a standard laboratory diet, CRF-1 (Oriental Yeast Co., Ltd., Tokyo, Japan), which contained L-methionine (0.45 g/100 g diets) and L-cystine (0.35 g/100 g diets) as sulfur amino acids (data provided by Oriental Yeast Co., Ltd.). All mouse studies were performed according to the animal experimental guidelines for laboratory issued by Animal Care Committee of Ajinomoto Co., Inc., and all of the experimental procedures were reviewed and approved by the Animal Care Committee of Ajinomoto Co., Inc. (approval number: 2017107 and 2,018,104).

### Administration of L-cystine and L-theanine

L-Cystine (Ajinomoto Co., Tokyo, Japan) was suspended in 0.5% methylcellulose. L-Theanine (Taiyo Kagaku Co., Ltd., Yokkaichi, Japan) was dissolved in 0.5% methylcellulose. Two hundred eighty milligrams per kilogram of L-Cystine and L-theanine suspension (CT: cystine 200 mg/kg, theanine 80 mg/kg) was administered orally to the mice. The control mice were administered 0.5% methylcellulose orally during the same period.

### Induction of intestinal mucositis

Intestinal mucositis was induced in male BALB/c mice by a single intraperitoneal injection of 5-FU (120–150 mg/kg). Two hundred eighty milligrams per kilogram of CT (cystine 200 mg/kg, theanine 80 mg/kg) or the equivalent volume of 0.5% methylcellulose was orally administered once daily, starting 3 days before 5-FU administration until the end of the experiment. Disease severity was evaluated daily by measuring body weight, food intake and the diarrhea score. Disease severity was scored using the following scale, 0: solid stool (Normal), 1: soft stool (Normal), 2: slightly wet and soft stool (Mild), 3: wet and unformed stool (Moderate), 4: watery stool (Severe).

### Small intestine histology

On days 1, 3, 4, 6, and 8 after 5-FU injection, mice were euthanized, and intestinal samples were collected, fixed with 4% paraformaldehyde overnight, and embedded in paraffin. The samples were cut into 5-μm thick sections, and stained with hematoxylin and eosin for histological analysis. The villus length and crypt depth were measured using Sensiv Measure Imager software (micro square Inc.) on images captured using an OLYMPUS BX61 microscope and an OLYMPUS DP71 camera (OLYMPUS). Ten intact and well-oriented villi and crypts were measured and averaged.

### Apoptosis analysis and proliferating cell nuclear antigen immunohistochemistry

Mice were sacrificed 1, 3, 4, 6 or 8 days after 5-FU injection, and intestinal tissue samples were collected, fixed with 4% paraformaldehyde overnight, and embedded in paraffin. The samples were cut into 5-μm thick sections. Apoptosis was detected by terminal deoxynucleotidyl transferase-mediated dUTP nick-end labelling (TUNEL) assay using the TaKaRa In Situ Apoptosis Detection Kit (TaKaRa: MK500). For each sample, the number of TUNEL-positive cells was counted from 10 crypts/mouse under a microscope, and the average value of 4–6 mice in each group was taken as the apoptosis index. Cell proliferation was determined immunohistochemically using the anti-proliferating cell nuclear antigen (PCNA) antibody (Sigma-Aldrich Inc., U.K.). The immunocomplex was visualized by TaKaRa POD conjugate anti-mouse, for mouse tissue. Sections were counter-stained with hematoxylin. The numbers of PCNA-positive cells were counted under a light microscope (BX-50, Olympus) from 10 crypts /mouse. The average number of PCNA-positive cells in each individual mouse was divided by the average number of PCNA-positive cells in normal mice and expressed as % PCNA.

### Measurement of GSH and GSSG levels in small intestinal tissue

The mice were randomly assigned to 3 groups: (1) a no treatment group (normal control group: Normal Control, *n* = 6), (2) a group receiving 150 mg/kg of 5-FU once (day 0) intraperitoneally (5-FU group: 5-FU, *n* = 6), or (3) a group receiving 150 mg/kg of 5-FU once (day 0) intraperitoneally and 280 mg/kg of CT once daily for 4 days (day − 3–day 0) orally (5-FU + CT group: 5-FU + CT, *n* = 6). Mice were sacrificed 18 h after 5-FU injection and the jejunum was collected. One hundred milligrams of the collected tissue was homogenized in 0.1 ml of 5% sulfosalicylic acid and centrifuged at 8000×g for 10 min. GSH and GSSG concentrations of the supernatants were measured using the GSSG/GSH Quantification Kit (Dojindo).

### Identification of ROS in the small intestine

Male Balb/c mice aged 7–9 weeks were subjected to the ligated intestinal loop experiment 18 h after 5-FU administration. The mice were fasted from the day before surgery, but they had access to water until immediately before surgery. Under inhalation anesthesia with 3% isoflurane (Mylan Inc.), midline laparotomy was performed and an intestinal loop with a 1-cm length was carefully prepared in the jejunum as to not to injure or ligate blood vessels. The Oxidative Stress Detection Reagent (2 μM, ROS-ID Total ROS/Superoxide Detection Kit Enzo) was dissolved in saline and 150 μL of the solution was injected into the loop by inserting a winged needle (27G × 1/2 0.40 × 13 mm TERUMO) into the intestinal lumen. After removing the injection needle, the intestine was ligated on the side close to the other ligation avoiding leakage of the solution from the hole made by the needle. Then, the skin incisions were closed using a suture staple. After leaving to rest for 1 h, the abdomen was re-opened, the small intestine was excised, and the loop region was taken out and washed with saline. The opened intestine was embedded in O.C.T. compound and 10-μm thin frozen sections were prepared. The sections were immediately observed under a fluorescence microscope and photographed, and 3 visual fields were selected per animal and ROS-positive crypts were counted.

### Anti-tumor efficacy of 5-FU in tumor-implanted mice

CT26 tumor (a murine colon cancer cell line created in Balb/c mice)- bearing mice were prepared by the following methods. CT26 cells (1 × 10^6^ each) were transplanted subcutaneously into the back of BALB/c mice under light isoflurane anesthesia and, 10 days later, these mice intraperitoneally received 5-FU at 150 mg/kg. From day 8 post transplantation, CT(280 mg/kg) or vehicle (Methylcellulose) was orally administered once daily for the consecutive 10 days. On day 18, mice were sacrificed and measured tumor weight.

### Statistical analyses

**Fig. 1:**
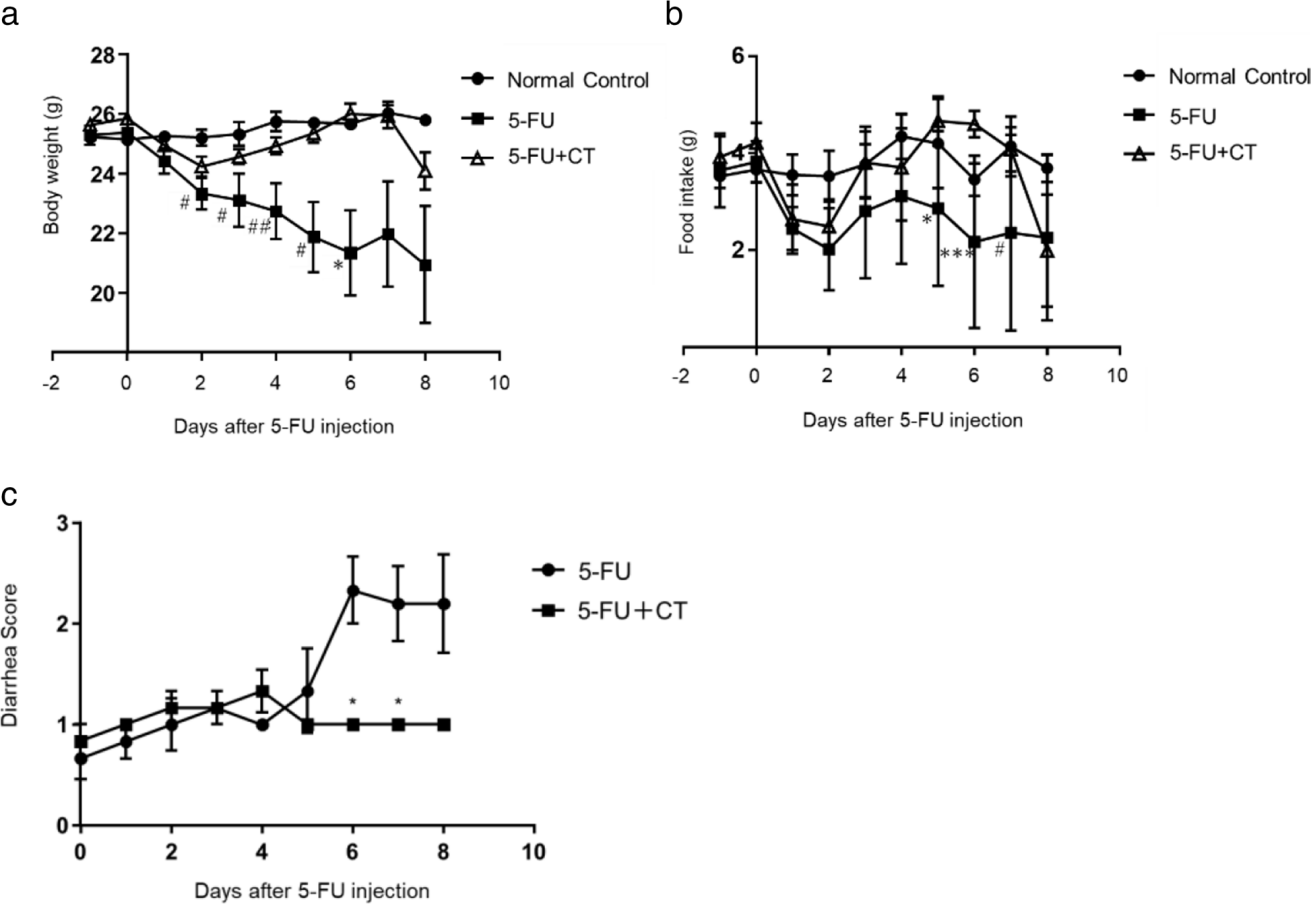
Effects of CT on body weight and food intake change and diarrhea development in 5-FU-induced small intestinal mucositis model mice. **a** Time-course changes in body weight. Values are expressed as the mean ± SEM. *N* = 5–6. #*p* < 0.05 vs normal control, ## *p* < 0.01 vs normal control, *p < 0.05 vs. 5-FU + CT by Dunn’s Multiple comparison tests **b**) Time course of changes in food intake. Values are expressed as the mean ± SEM. *N* = 5–6. #*p* < 0.05 vs normal control, *p < 0.05 vs. 5-FU + CT, ***p < 0.001 vs. 5-FU + CT by Dunn’s multiple comparison test **c)** Diarrhea score: Diarrhea symptoms were observed every day and the severity of diarrhea was scored using the following scale, 0: solid stool (Normal), 1: soft stool (Normal), 2: slightly wet and soft stool (Mild), 3: wet and unformed stool (Moderate), 4: watery stool (Severe). Values are expressed as the mean ± SEM. N = 5–6. * *p* < 0.05, compared with 5-FU by the Mann-Whitney U test

**Fig. 2:**
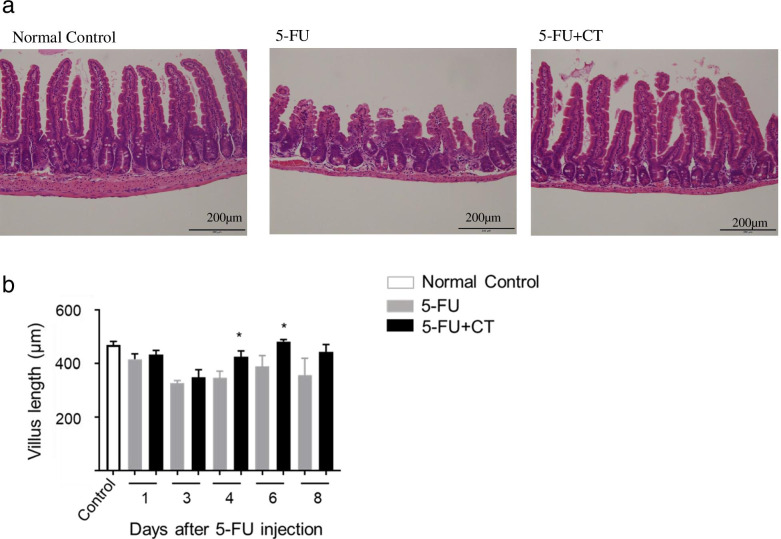
Small intestinal villus-protecting effects of CT in 5-FU-induced small intestinal mucositis model. **a** Typical small intestinal villus tissue in each group. The tissue was excised on day 6 after 5-FU administration and subjected to hematoxylin-eosin staining. **b** Time-course changes in small intestinal villus length (from the top of villus to the villus-crypt junction). H/E-stained small intestinal villus tissue was observed under a light microscope. Ten villi with relatively normal morphology were selected per animal, the villus length was measured, and the mean was regarded as individual mice data. Values are expressed as the mean ± SEM. *N* = 5–6. *p < 0.05 vs 5-FU by the two-tailed Student’s t-test

**Fig. 3:**
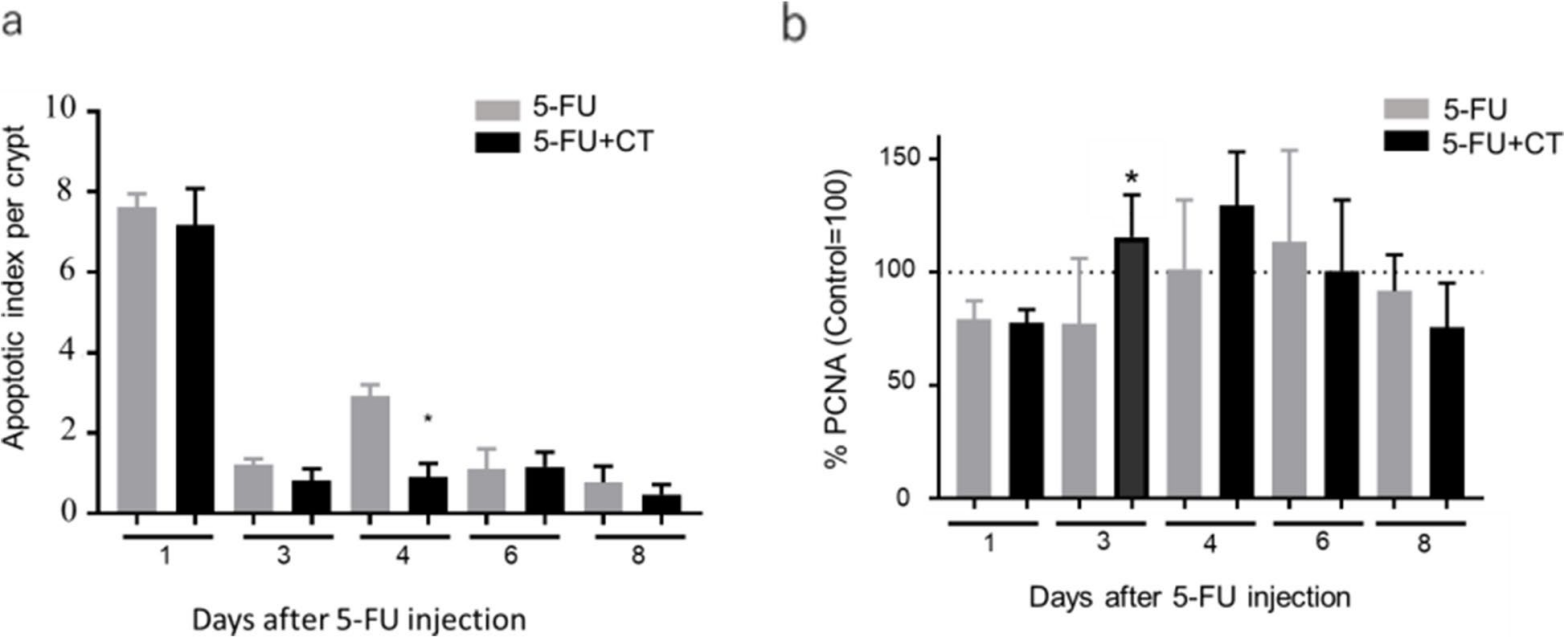
Effects of CT on the induction of apoptosis and proliferation of small intestinal crypt cells in 5-FU-induced small intestine mucositis model mice. **a** Apoptosis was evaluated by the TUNEL assay. TUNEL-positive cells were counted in 10 small intestinal crypts per specimen under a light microscope. Values are expressed as the mean ± SEM. *N* = 5–6. * *p* < 0.05 vs 5-FU by the two-tailed Student’s t-test. **b** Cell proliferation activity was immunohistochemically measured using the anti-PCNA antibody. PCNA-positive cells were counted in intestinal crypts under a light microscope. The average number of PCNA-positive cells in each individual mouse was divided by the average number of PCNA-positive cells in normal mice and expressed as % PCNA. Values are expressed as the mean ± SEM. *N* = 5–6. * *p* < 0.05 vs 5-FU by the two-tailed Student’s t-test

**Fig. 4:**
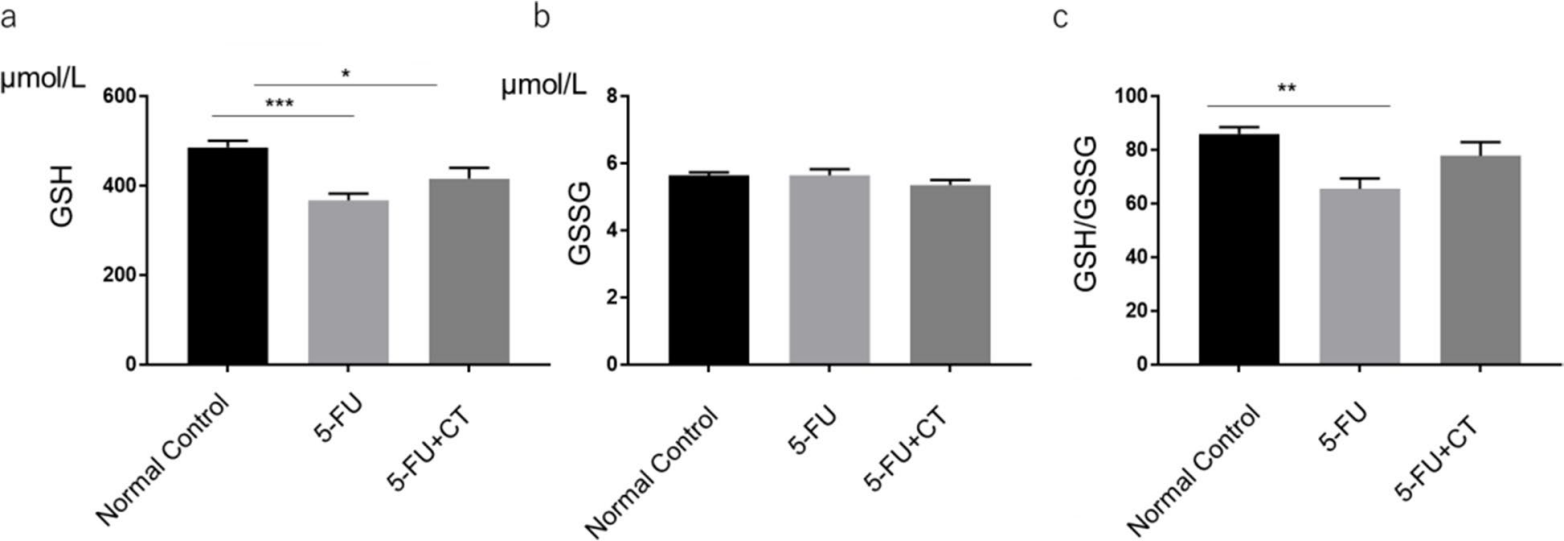
Protection against 5-FU-induced small intestinal GSH decrease by CT administration. **a** Small intestinal GSH level, **b** small intestinal GSSG level, and **c** small intestinal GSH/GSSG ratio are shown. Normal Control, control group receiving saline. 5-FU, receiving 5-FU (150 mg/kg) and methyl cellulose (the vehicle for CT). 5-FU + CT, receiving 5-FU (150 mg/kg) and CT (280 mg/kg). Values are expressed as the mean ± SEM. *N* = 5–6. *p < 0.05 vs normal control, **p < 0.01 vs normal control, ***p < 0.001 vs normal control by Tukey’s multiple comparisons test

**Fig. 5:**
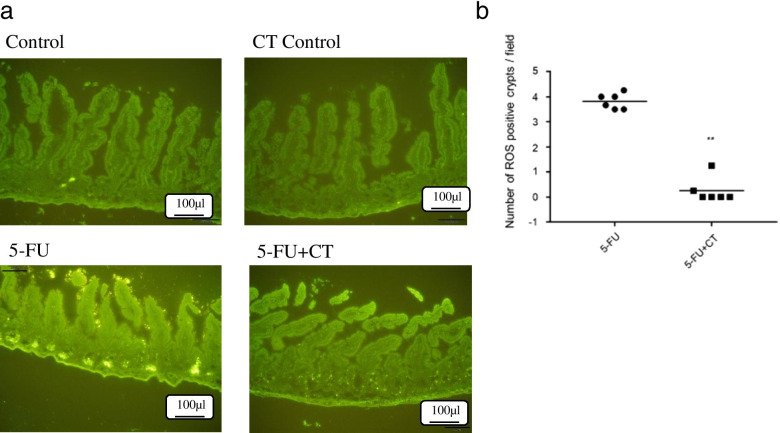
CT suppressed 5-FU induced ROS production in the small intestine. **a** Fluorescence histochemical study of ROS in intestinal mucosa using ROS-specific fluorescent probe. Control, receiving saline (the vehicle for 5-FU) and methyl cellulose (the vehicle for CT). CT Control, receiving saline (the vehicle for 5-FU) and CT (280 mg/kg). 5-FU, receiving 5-FU (150 mg/kg) and methyl cellulose (the vehicle for CT). 5-FU + CT, receiving 5-FU (150 mg/kg) and CT (280 mg/kg). **b** Combined data from three experiments showing the numbers of ROS-positive crypts. The numbers of positive crypts in the intestine were counted using a fluorescence microscope. After counting the ROS-positive crypts in 3 visual fields per mouse, the count number (0) in the control group was subtracted and the average value was used as the number for each mouse. ROS-positive crypts were counted in 3 visual fields per mouse and the mean was adopted as data of individual mice. Each data point represents an individual mouse and bars represent the mean. ** *p* < 0.01 5-FU vs 5-FU + CT by Mann Whitney test

**Fig. 6:**
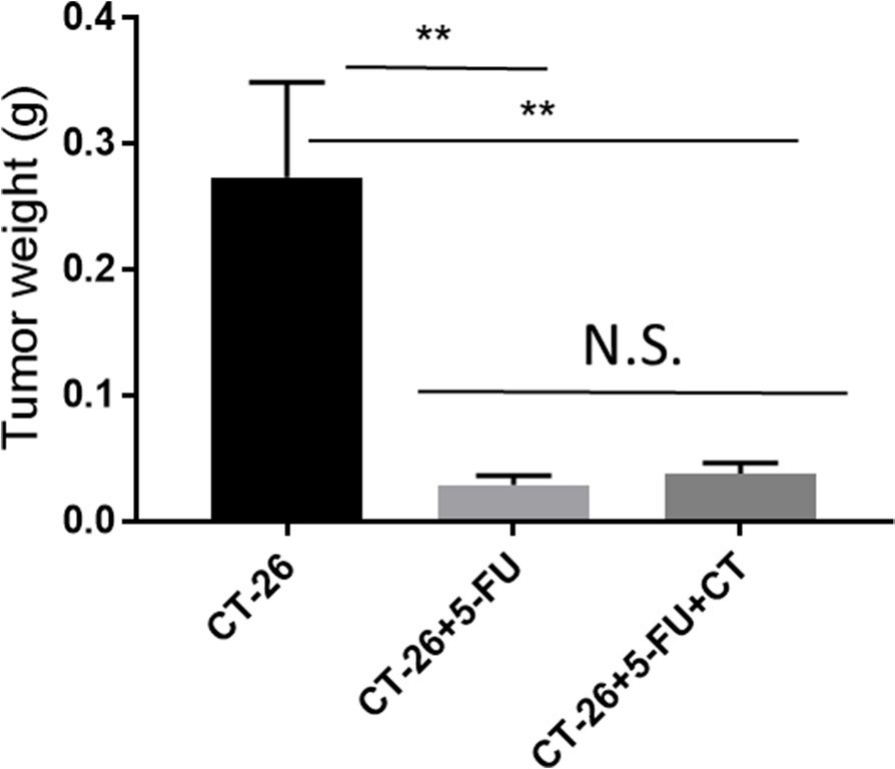
Influence of CT on the antitumor efficacy of antitumor drug in a tumor-bearing mouse model. CT26 murine colon cancer cells (1 × 106) were subcutaneously transplanted into male BALB/c mice. From Day 8 post transplantation, mice were orally administered CT (280 mg/kg) or methyl cellulose (the vehicle for CT) once daily for 10 days. The mice received 5-FU (150 mg / kg, i.p.) on day 10 after transplantation. The data is presented as the mean ± SEM of 6 mice. CT26 group vs CT26 + 5-FU and CT26 group vs CT26 + 5FU + CT group: ** < 0.01. CT26 + 5-FU group vs CT26 + 5-FU + CT group: No significant difference. (Dunnet’s multiple composition test)

All data were expressed as the mean ± SEM. The Prism version 7.05 software package (Graph pad software, CA, USA) was used for a two-tailed Student’s t-test (Fig. 2b and Fig. 3a,b), one-way ANOVA followed by the Tukey test (Fig. 4), Mann-Whitney test (Fig. 1b and Fig. 5b), Dunn’s test (Fig. 1a), or Dunnett’s test (Fig. 6) as a multiple comparison test, with *P*-values < 0.05 regarded as significant.

## Results

In the 5-FU group, body weight loss occurred already the day after treatment administration; the mean body weight decreased by approximately 82.3% (from 25.4 g to 20.9 g) by day 8 of the study. The amount of food intake also started to decrease the day after the administration of 5-FU. After that, there was a tendency to increase 3 days after the administration of 5-FU, but it did not reach the normal level until the end of the experiment. Diarrhea was observed from day 5 after 5-FU administration; the diarrhea score reached a plateau (mean score, 2.25 ± 0.86) between days 6 and 8. CT (280 mg/kg) was orally administered once daily, starting 3 days before the administration of 5-FU. In the 5-FU + CT group, the rate of body weight loss tended to improve after day 3 from the administration of 5-FU and after day 6, a statistically significant difference of body weight loss was found compared with the 5-FU group (*p* < 0.05; Fig. 1a). Food intake decreased in the 5-FU + CT administration group as well, but it showed an improvement tendency from 3 to 4 days after 5-FU administration and from days 5 to 6, a statistically significant difference in food intake was found between the 5-FU + CT group and the 5-FU group (*p* < 0.05; Fig. 1b). Concurrently, the diarrhea score did not increase in the CT treatment group after day 6; and, a statistically significant difference was observed between the treatment groups on days 6 and 7 (both *p* < 0.05; Fig. 1b).

### CT impact on 5-FU-induced damage in the small intestine

The small intestine villus on day 6 after 5-FU (120 mg/kg) administration is shown in Fig. 2a. The villus length of the 5-FU group was substantially shorter than that of the normal control group; however, the degree of this shortening was relatively reduced in the 5-FU + CT group. Time-dependent changes in the length of the small intestine villi are shown in Fig. 2b. The length of the villi in the 5-FU group was approximately 30% shorter than that in the control group on day 3; although it subsequently increased, it failed to return to its baseline length (mean of normal control). Similarly, in the 5-FU + CT group, the length of the intestinal villi decreased by 24% on day 3, which was similar to the decrease observed in the 5-FU group; however, the villi recovered to 86 and 100% of their baseline length (mean of normal control) on days 4 and 6, respectively. The small intestine villus length of the 5-FU + CT group was significantly longer than that of the 5-FU group on days 4 and 6 (both *p* < 0.05).

### Impact of CT on the small intestine crypt cell apoptosis and proliferation

In our experiments, on days 1 and 4 following 5-FU administration, TUNEL-positive cells were observed in the 5-FU group. In addition, the number of TUNEL-positive cells observed in this group was greater on day 1 than on day 4. Meanwhile, the number of TUNEL-positive cells in the 5-FU + CT group was not significantly different from that in the 5-FU group on day 1; however, it significantly decreased on day 4 (*p* < 0.05; Fig. 3a). In the 5-FU group, the mean number of PCNA-positive cells decreased by approximately 76.6% from the mean of the normal control on the day after 5-FU administration and then recovered to be greater than that of the control group on day 6. Similarly, in the 5-FU + CT group, the mean number of PCNA-positive cells decreased by 75.2% of that of the control group the day after treatment, which was similar to the decrease observed in the 5-FU group; however, the number in the 5-FU + CT group recovered to 118.4 and 125.7% on days 3 and 4, respectively. The number in the 5-FU + CT group on day 3 was significantly higher than that in the 5-FU group (*p* < 0.05; Fig. 3b).

### Impact of CT on glutathione production in the small intestine

The levels of oxidized and reduced glutathione levels (GSSG and GSH, respectively) in the small intestine villi were measured 18 h after 5-FU administration. In the 5-FU group, the GSH level was significantly lower than that in the normal control group (*p* < 0.001). This reduction in GSH level tended to recover with CT administration (Fig. 4a). However, no significant differences were observed in GSSG levels among the groups (Fig. 4b). While as shown in Fig. 4c, 5-FU administration significantly decreased the GSH-GSSG ratio compared to the normal control group (*p* < 0.01), CT treatment returned it to its normal control level.

### Impact of CT on ROS production in the small intestine

To investigate the influence of CT on ROS production in the small intestine, tissue ROS was visualized using an ROS-specific fluorescent probe. In the 5-FU group, ROS-positive crypts were observed in the basal region of crypts 18 h after administration. On the other hand, no ROS-positive crypts were observed in the control group and CT control group in which CT was administered to normal mice. (Fig. 5a). In the 5-FU + CT group, the number of ROS-positive crypts were decreased compared with that in the 5-FU group (*p* < 0.01), suggesting that CT induced the scavenging of ROS (Fig. 5b).

### Impact of CT on 5-FU antitumor effects

The induction of oxidative stress, including the production of ROS, in tumor tissue contributes to the antitumor effects of several anticancer drugs; thus, the effect of CT administration on the antitumor action of 5-FU was investigated using a subcutaneous transplant model of CT26, which is a mouse colon cancer. The tumor weight was significantly reduced in the 5-FU and in the 5-FU + CT groups compared with the CT26 transplanted control group (both *p* < 0.01; Fig. 6). However, no significant difference in tumor weight was observed between the 5-FU and 5-FU + CT groups.

## Discussion

Our study showed that CT administration reduced diarrhea in a mouse 5-FU-induced mucositis model. The results of our study are consistent with our previous human studies, CT broadly alleviate the adverse effects of chemotherapy, including diarrhea in cancer patients, thereby reducing the incidence of withdrawal / discontinuation of chemotherapy and increasing the treatment completion rate [22]. CT administration reduced the shortening of small intestinal villi and the destruction of crypto, which are the morphological features of 5-FU-induced mucositis. In addition, CT administration improved the GSH / GSSG ratio to normal control levels and inhibited 5-FU-induced ROS production in intestinal tissue. This suggests that CT administration suppressed mucositis by improving the redox state, resulting in suppression of diarrhea.

In the mucositis model used in this study, 5-FU administration induced and inhibited villus stem cell apoptosis and proliferation, respectively, resulting in the shortening of the small intestine villus length. Body weight loss occurred already on the day following 5-FU administration, suggesting that its underlying mechanism is associated with 5-FU toxicity rather than diarrhea. On the other hand, in the 5-FU + CT administration group, the body weight began to increase from the 3rd day of 5-FU administration and then recovered to the normal level. Food intake decreased from the day after administration of 5-FU. Food intake also decreased in the 5-FU + CT administration group, but there was a tendency for improvement from days 3 to 4 after 5-FU administration. From days 5 to 6, the food intake was significantly higher than that in the 5-FU administration group. This phenomenon coincided with the change in body weight over time (Fig. 1a, b). However, it was before the onset of diarrhea and was thought to be unrelated to the diarrhea-suppressing effect of CT (Fig. 1b).

Diarrhea was observed in the mice in the 5-FU administration group on the 6th day of administration, but no diarrhea symptoms were observed in any of the mice in the 5-FU + CT group. This result indicates that CT suppresses 5-FU-induced diarrhea symptoms (Fig. 1c). We focused on the promotion glutathione production by CT and the improvement in the intestinal redox environment from oxidized to reduced. The gut microbiota is one of the factors that determines the intestinal environment, including the redox state. Although gut microbiota are possible targets for CT, we have not yet investigated the effects of CT administration on enterobacteria but will do so in future studies. The intestinal villus length and crypt cell proliferation rate decreased from the day after 5-FU administration, with and without CT administration. The recovery of the small intestine villus length on day 4 in the 5-FU + CT group was consistent with the improved rate of cell proliferation observed on day 3. In particular, as shown in Fig. 3b, the % PCNA value of the 5-FU + CT group exceeded that of the normal control tissue on days 3 and 4. This suggests the presence of hyperplasia associated with oxidative colitis [23]. PCNA is a proliferation marker that changes almost in tandem with Ki67 and shows hyperproliferation in crypts [24]. PCNA is also a marker of DNA repair and shows enhanced repair against DNA damage caused by 5-FU. The early increase in PCNA-positive cells in the 5-FU + CT group suggests that repair and regeneration began early due to CT. From these results, ROS production by administration of 5-FU caused oxidative stress in crypts of small intestinal villi, and induction of apoptosis and suppression of growth due to DNA damage were observed. In the 5-FU + CT group, the damage to crypts caused by oxidative stress was alleviated, and the shortening of villus length was alleviated by suppressing ROS production. The results in Fig. 5a suggested the possibility that the signal-positive cells that concentrate on the bottom of crypts and showed ROS production were Paneth cells. Paneth cells are a kind of neutrophil cells. When foreign substances invade crypts, Paneth cells release ROS to protect crypt base columnar (CBC) stem cells. CBC cell division/proliferation requires contact with Paneth cells. Considering these facts, it is quite possible that the effect of CT on the activity of Paneth cells was one of the factors underlying the protective effect on small intestinal villi by CT administration.

5-FU is an antimetabolite anticancer drug, while the inhibition of DNA/RNA synthesis is considered the key factor in the induction of apoptosis. Recently, it has been reported that 5-FU-induced ROS production leads to apoptosis [25]. In our study, 5-FU increased the number of apoptotic cells in the intestinal crypt on days 1 and 4, but CT administration did not suppress apoptosis on day 1. In fact, CT could not suppress apoptosis in the acute phase mediated by apoptosis-related factors such as inhibited DNA/RNA synthesis. In contrast, the second peak of the number of apoptotic cells that appeared on day 4 was suppressed by CT administration. Since the contribution of 5-FU remaining in the body is expected to decrease on day 4 after 5-FU administration, the apoptosis observed on day 4 might have occurred due to the effect of ROS rather than due to the direct effect of 5-FU. These results are supported by a previous report showing that the second peak of the number of 5-FU-induced apoptosis observed on day 3 was significantly lower in Nox1KO mice in which ROS production was suppressed by Nox1 deficiency [25].

In the present study, GSH and GSSG levels were measured at 12 to 24 h after 5-FU administration. 5-FU administration reduced GSH levels in the small intestine tissue, where it tended to increase with CT administration. In addition, CT administration returned the GSH/GSSG ratio to normal levels. The ratio of GSH to GSSG greatly affects the intracellular redox potential (proportional to the logarithm of [GSH] 2 / [GSSG]) [26]. When oxidative stress exceeds the cell’s ability to reduce GSSG to GSH, GSSG is either actively excreted from the cell or reacts with the protein sulfhydryl group to cause the formation of mixed disulfides to prevent a major shift in redox equilibrium. Therefore, repeated or severe oxidative stress depletes cellular GSH [27]. In addition, oxidative stress activates a system called NRF-2/Keep countering the antioxidant stress of cells. These include glutathione-S-transferase (GST), which directly detoxifies electrophiles, and glutamylcysteine synthase, which synthesizes depleted glutathione [28]. Administration of CT means that such redox balance shifts to the oxidized form and the antioxidant system is activated to supply constituents of glutathione to promote glutathione synthesis. Kawashiri et al. reported that repeated doses of cystine and theanine (equivalent to CT) increased the total GSH levels in the sciatic nerve in a rat oxaliplatin-induced peripheral neuropathy model [29]. In addition, Kurihara et al. found that CT administration increased the production of antigen-specific IgG antibodies using a mouse immunization model. They reported that the values of total GSH and the GSH/GSSG ratio of the liver decreased by immunization were significantly increased by CT administration [8]. We also found an increase in total GSH concentration in the villous tissue of the small intestine in a 5-FU-induced enteritis model (data not shown). Unfortunately, no significant increase in reduced GSH was observed (*p* = 1.17). It could be that GSH produced in inflamed tissue was immediately oxidized to GSSG, which was actively excreted from cells or reacted with the protein sulfhydryl group to cause the formation of mixed disulfides.

ROS are produced during oxidative processes such as glycolysis in the cytosol, the citric acid cycle in mitochondria and oxidative phosphorylation. In particular, mitochondrial redox reactions are a major source of ROS in most cells, including tumor cells [28]. ROS are produced in small amounts in the body and are involved in cell regulation, homeostatic maintenance, and functions such as signal transduction, gene expression, and receptor activation [30]. However, excess production of ROS causes cell dysfunction such as loss of energy metabolism, disruption of cell signaling, gene mutations, tumorigenesis, immune activation, and inflammation [31, 32]. In such cases, it is known that NRF2 migrates to the nucleus and activates antioxidative target genes to restore the redox balance [28]. One of the results is that the gene expression of the GSH synthase group was enhanced, and the GSH synthesis was promoted. GSH plays an important role in controlling the cellular level of ROS [32, 33]. In patients with viral infections and chronic inflammation, a decrease in the amount of GSH in the body, which is considered to be depleted of GSH, is observed, and this tendency is particularly marked in severely ill patients. In a mouse asthma model, NAC, a thiol with antioxidant properties and one of the substrates in GSH biosynthesis, replenishes the GSH pool by its administration, restores the redox balance, and suppresses allergen-induced airway-reactive inflammation [34]. In addition, it has been reported in human studies that supplementation with NAC restores glutathione synthesis and the in vivo GSH levels and has the effect of reducing oxidative stress in diseases associated with aging and low GSH levels [35]. In this study, CT administration, that is, administration of cystine and theanine, which are two amino acids that make up glutathione, promoted GSH synthesis and normalized the redox balance. As a result, CT administration suppressed enteritis, a side effect of the anticancer drug 5-FU, and alleviated diarrhea symptoms. In the present study, we used a ROS-specific fluorescent probe to investigate ROS production as early as 18 h after 5-FU administration. After 5-FU administration, a strong ROS signal-positive crypt was detected in the mucosal tissue of the small intestine. These signals were reduced by CT administration, suggesting that CT may inhibit ROS production. The suppression of cell dysfunction associated with reduced ROS production and concurrent tissue protection might be the mechanisms underlying CT mucositis and diarrhea symptom reduction. Sonis’s a five-stage process of pathobiology of mucositis supports our data and ideas. They proposed the biological sequence of mucositis can be divided into five phases including initiation, signal transduction, amplification, ulceration, and healing, and these biological events occur sequentially [36, 37]. In addition, chemotherapy-induced DNA damage and ROS production are two characteristic events at the initiation phase [38, 39] and suppressing the initiation step is most effective in reducing mucositis [40]. CT might be thought to have suppressed mucositis by suppressing the production of ROS and stopping the biological sequence of mucositis at the initiation phase. Furthermore, we hypothesis that the attenuation of ROS production by CT administration may have suppressed the progression to the second and third steps of the five-stage process. However, further studies should examine the inhibitory effects of CT on the activation of transcription factor and inflammatory cytokine production in this process to verify this hypothesis.

The antitumor effect of 5-FU is inhibition of DNA and RNA synthesis, and it is thought that the promotion of glutathione synthesis and the improvement of the redox environment by administration of CT do not directly affect the antitumor effect of 5-FU. On the other hand, suppression of cancer cell proliferation by inhibiting DNA and RNA synthesis causes ROS production, and the produced ROS are cytotoxic to cancer cells. Therefore, it is often considered that CT administration, which promotes the production of GSH, an antioxidant, may suppress the antitumor effect of anticancer drugs. However, using the CT-26 cell subcutaneous transplant model, we confirmed that CT itself did not affect the proliferation of CT-26 cells (Additional file 1: Fig. S1) and that CT administration had no effect on the antitumor effect of 5-FU (Fig. 6). We also confirmed that CT did not suppress the antitumor effect of cisplatin in a model of subcutaneous transplantation of PC3 cells (Additional file 2: Fig. S2). Furthermore, Kawashiri et al. reported that CT did not affect the antitumor effect of oxaliplatin in a C-26 cell subcutaneous transplantation model. Cancer cells maintain abnormal growth and growth rates compared to noncancer cells, require high energy levels and undergo excessive cellular metabolism. Therefore, ROS production by mitochondria and NADPH oxidase is increased, which upregulates the activity of the antioxidant system. In other words, the antioxidant system of cancer cells is different in scale from that of normal cells [33, 41]. It is possible that the amount of CT used in this experiment was sufficient for the antioxidant system of normal cells but too small to be effective in cancer cells.

## Conclusion

In conclusion, our results indicate that CT administration suppresses 5-FU-induced mucositis by improving the glutathione redox environment and inhibiting ROS production in the small intestine, resulting in the alleviation of diarrhea. Concurrently, CT does not reduce the antitumor effects of 5-FU. Overall, our results suggest that the intake of CT, the source of antioxidant GSH, may be a promising supportive care for patients undergoing chemotherapy.

## Supplementary Information

**Additional file 1: Figure S1.** Influence of CT on tumor growth in a tumor-bearing mouse model. CT26 murine colon cancer cells (1 × 10^6^) were subcutaneously transplanted into male BALB/c mice. From day 8 post transplantation, mice were orally administered CT (280 mg/kg) or methyl cellulose (the vehicle for CT) once daily for 10 days. The data are presented as the mean ± SEM of 6 mice. CT26 group vs CT26 + CT group: No significant difference by the two-tailed Student’s t-test.

**Additional file 2: Figure S2.** Influence of CT on the antitumor efficacy of antitumor drugs in a tumor-bearing mouse model. We investigated the influence of CT administration in a mouse model with subcutaneously transplanted human prostate cancer cells (PC-3) using cisplatin (CDDP). Oxidative stress plays an important role in its anticancer action compared with that of 5-FU. As shown in Fig. S2, no difference was noted in the tumor growth rate between the control and CT treatment groups. In addition, a similar degree of antitumor activity was noted in the CDDP anticancer drug treatment group and anticancer drug +CDDP+CT treatment group. These findings confirm that CT does not influence tumor proliferation or the antitumor effects of CDDP, an anticancer drug. PC-3 vs CT280: No significant difference (two-tailed Student’s t-test). PC-3 vs CDDP: * < 0.05 (two-tailed Student’s t-test). CDDP vs CDDP+CT70, CDDP+CT140, and CDDP+CT280: No significant difference. (Dunnet’s multiple composition test).

## Data Availability

The datasets used and/or analyzed during the current study available from the corresponding author on reasonable request.

## Abbreviations

ROS: Reactive oxygen species
5-FU: 5-fluorouracil
CT: Cystine and theanine
GSH: Glutathione
GSSG: Glutathione-S-S-Glutathione
TUNEL: TdT-mediated dUTP Nick End Labeling
TS: Thymidylate synthase
PCNA: Proliferating cell nuclear antigen
HFS: Hand foot syndrome
