# Supplementary material for: Oral administration of cystine and theanine attenuates 5-fluorouracil-induced intestinal mucositis and diarrhea by suppressing both glutathione level decrease and ROS production in the small intestine of mucositis mouse model
Source: BMC Cancer. 2021 Dec 18;21:1343. doi: 10.1186/s12885-021-09057-z (PMC8684148; doi:10.1186/s12885-021-09057-z)
Supplement: Supplementary file 1 — Additional file 1: Figure S1. Influence of CT on tumor growth in a tumor-bearing mouse model. CT26 murine colon cancer cells (1 × 10^6^) were subcutaneously transplanted into male BALB/c mice. From day 8 post transplantation, mice were orally administered CT (280 mg/kg) or methyl cellulose (the vehicle for CT) once daily for 10 days. The data are presented as the mean ± SEM of 6 mice. CT26 group vs CT26 + CT group: No significant difference by the two-tailed Student’s t-test. [file 12885_2021_9057_MOESM1_ESM.pptx]

## Slide 1
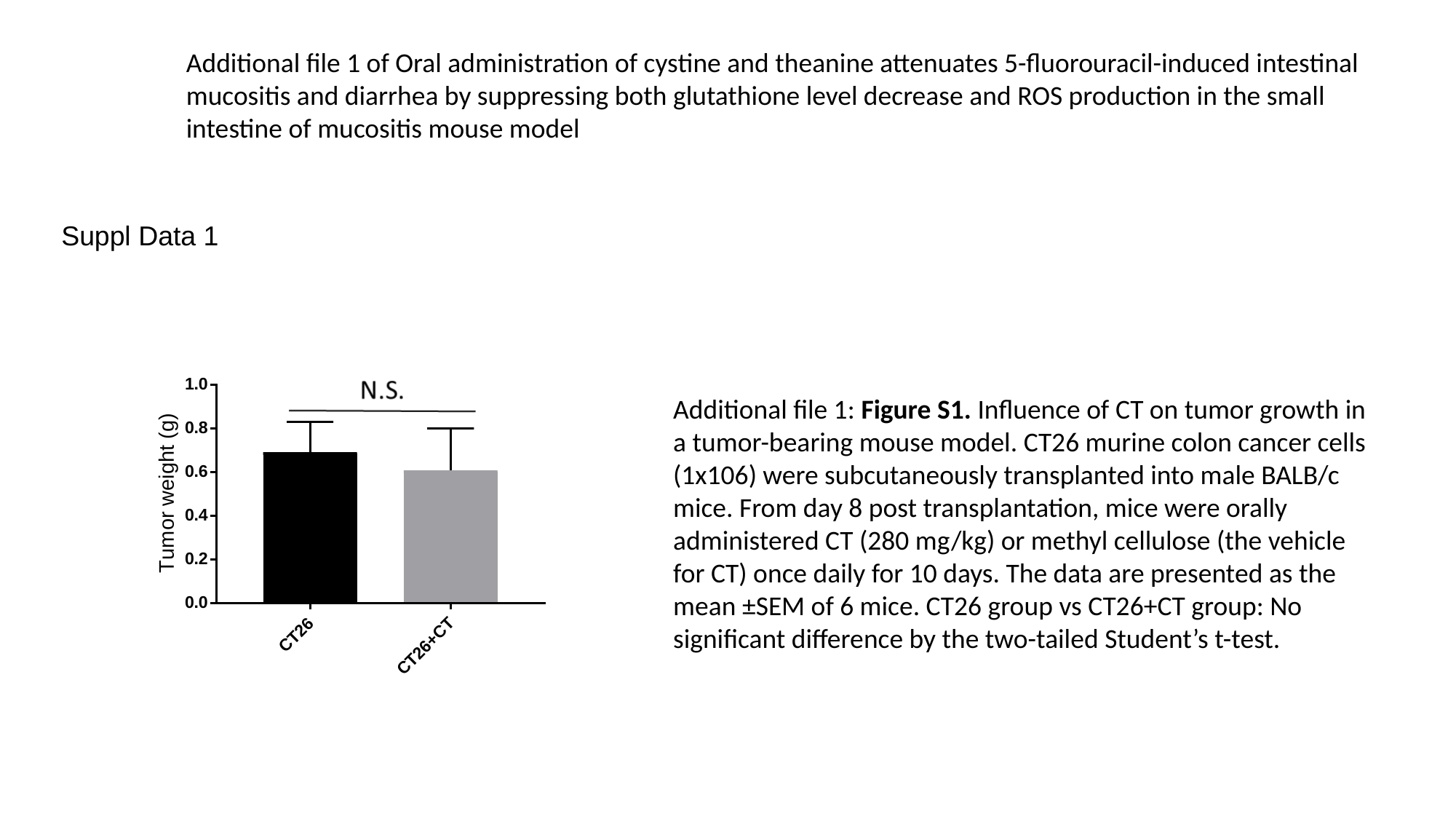

Additional file 1 of Oral administration of cystine and theanine attenuates 5-fluorouracil-induced intestinal
mucositis and diarrhea by suppressing both glutathione level decrease and ROS production in the small
intestine of mucositis mouse model
Suppl Data 1
Additional file 1: Figure S1. Influence of CT on tumor growth in a tumor-bearing mouse model. CT26 murine colon cancer cells (1x10^6^) were subcutaneously transplanted into male BALB/c mice. From day 8 post transplantation, mice were orally administered CT (280 mg/kg) or methyl cellulose (the vehicle for CT) once daily for 10 days. The data are presented as the mean ±SEM of 6 mice. CT26 group vs CT26+CT group: No significant difference by the two-tailed Student’s t-test.
