# Supplementary material for: Oral administration of cystine and theanine attenuates 5-fluorouracil-induced intestinal mucositis and diarrhea by suppressing both glutathione level decrease and ROS production in the small intestine of mucositis mouse model
Source: BMC Cancer. 2021 Dec 18;21:1343. doi: 10.1186/s12885-021-09057-z (PMC8684148; doi:10.1186/s12885-021-09057-z)
Supplement: Supplementary file 2 — Additional file 2: Figure S2. Influence of CT on the antitumor efficacy of antitumor drugs in a tumor-bearing mouse model. We investigated the influence of CT administration in a mouse model with subcutaneously transplanted human prostate cancer cells (PC-3) using cisplatin (CDDP). Oxidative stress plays an important role in its anticancer action compared with that of 5-FU. As shown in Fig. S2, no difference was noted in the tumor growth rate between the control and CT treatment groups. In addition, a similar degree of antitumor activity was noted in the CDDP anticancer drug treatment group and anticancer drug +CDDP+CT treatment group. These findings confirm that CT does not influence tumor proliferation or the antitumor effects of CDDP, an anticancer drug. PC-3 vs CT280: No significant difference (two-tailed Student’s t-test). PC-3 vs CDDP: * < 0.05 (two-tailed Student’s t-test). CDDP vs CDDP+CT70, CDDP+CT140, and CDDP+CT280: No significant difference. (Dunnet’s multiple composition test). [file 12885_2021_9057_MOESM2_ESM.pptx]

## Slide 1
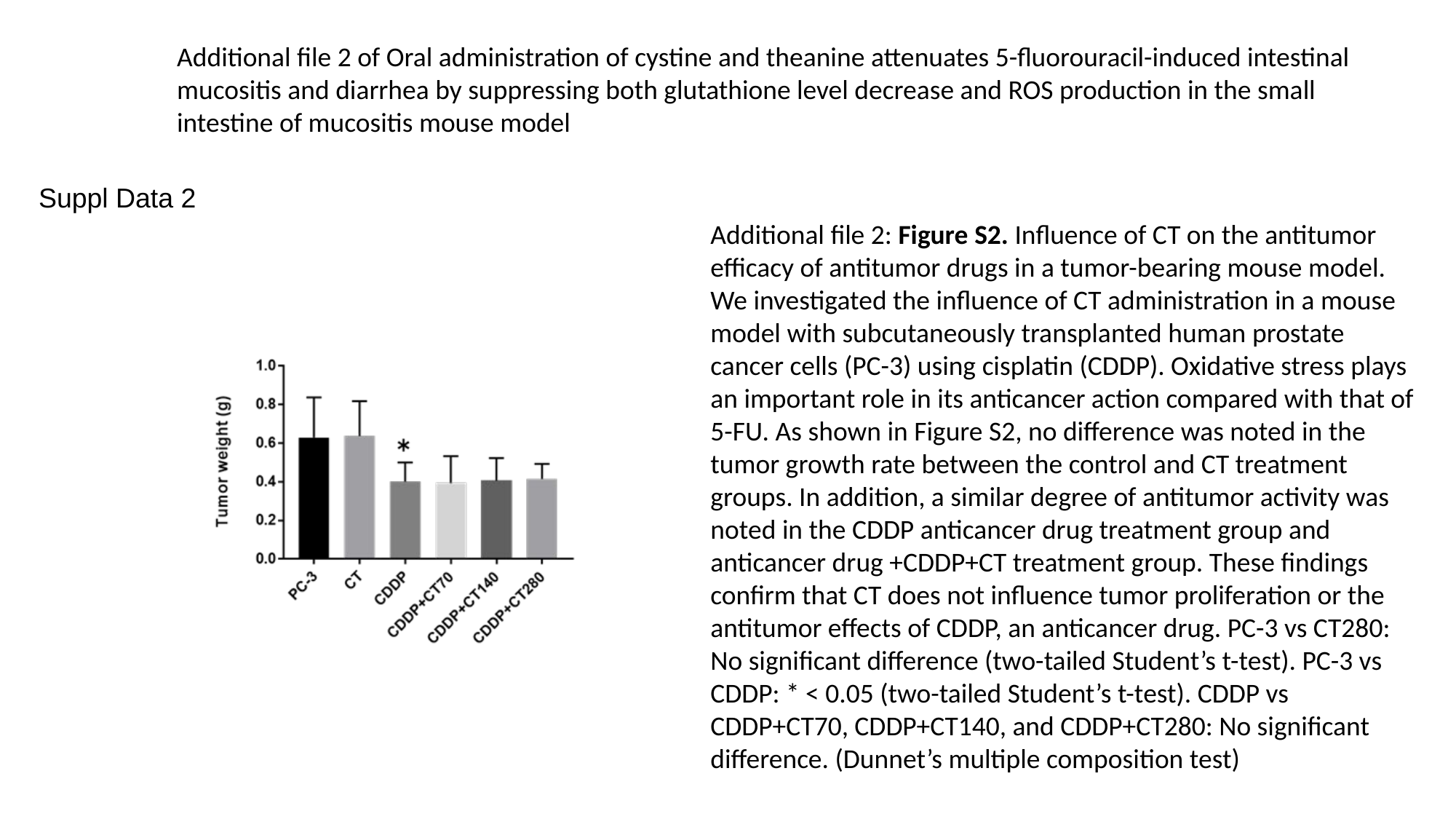

Additional file 2 of Oral administration of cystine and theanine attenuates 5-fluorouracil-induced intestinal
mucositis and diarrhea by suppressing both glutathione level decrease and ROS production in the small
intestine of mucositis mouse model
Suppl Data 2
Additional file 2: Figure S2. Influence of CT on the antitumor efficacy of antitumor drugs in a tumor-bearing mouse model. We investigated the influence of CT administration in a mouse model with subcutaneously transplanted human prostate cancer cells (PC-3) using cisplatin (CDDP). Oxidative stress plays an important role in its anticancer action compared with that of 5-FU. As shown in Figure S2, no difference was noted in the tumor growth rate between the control and CT treatment groups. In addition, a similar degree of antitumor activity was noted in the CDDP anticancer drug treatment group and anticancer drug +CDDP+CT treatment group. These findings confirm that CT does not influence tumor proliferation or the antitumor effects of CDDP, an anticancer drug. PC-3 vs CT280: No significant difference (two-tailed Student’s t-test). PC-3 vs CDDP: * < 0.05 (two-tailed Student’s t-test). CDDP vs CDDP+CT70, CDDP+CT140, and CDDP+CT280: No significant difference. (Dunnet’s multiple composition test)
